# On the Diseases of Children

**Published:** 1830-04-01

**Authors:** 


### On the Diseases of Children. By Mr. Marley.

Mr. Marley, though a young surgeon, appears to be an attentive observer, and a judicious practitioner. It is perhaps to be regretted that he has ushered himself so early on the stage of professional literature. Medical knowledge, like wine, gains by keeping. Gross errors fall to the bottom, and the lighter ones float on the top, so that both may be separated from the purified fluid between. We do not address this remark to the author of the present work in particular, but to our junior brethren generally. It will not be attended to, and therefore we shall not press the subject farther. We shall glance at some of the sections in this volume, by way of offering our readers some specimens of the performance.

### Practical Remarks on the Use of Opium.

"In children labouring under severe abdominal pain from an irritable state of the intestinal canal, we often find an appropriate dose of opium (it will be understood that I mean any of its preparations,) given either in form of draught or enema, produce beneficial and speedy relief. The surface, which was before dry and parched, becomes moist, and is succeeded by a gradual cessation of pain, and probably by a sound and undisturbed sleep. But this picture is sometimes reversed, for instead of being quieted, the child will start up suddenly, screaming out as if frightened, or he will moan during a restless and imperfect slumber. When opiates produce the latter train of symptoms, I have generally observed, that on the occurrence of slight diaphoresis, the patient becomes tranquillized, and a calm and quiet sleep will often follow. The warm bath will be found of great utility by producing slight moisture on the surface, and should therefore be employed with that view.

"The power of opiates, in allaying irritation, is probably nowhere more marked and efficacious than in excessive evacuations from the bowels. In such cases it is in general best to exhibit it in the form of enema; but even in this form caution should guide us in its use. In one instance I have known an injection, containing a very small quantity of laudanum, produce great cerebral excitement, extreme thirst and vomiting.

"In those cases of excitement arising from nervous irritability, its well-timed use will often prove decisive. After bleeding in inflammation of the bowels, opiates will often be found of great use, and should be exhibited per anum. In colic pains they prove highly efficacious, and should never be neglected."

Mr. M. properly considers opium as contra-indicated in affections of the lungs, where there is dry cough and quick pulse—also in all cases of increased action of the brain or its membranes. The author conceives, that "the lives of many children are annuall sacrificed by the indiscriminate and improper use of opiates." He instances some cases where mischief was produced by the exhibition of opium to children, and indeed such instances are by no means rare—especially where quack medicines are employed. In truth there is rarely any necessity for the exhibition of opiates to children, excepting in some severe bowel complaints, and then they should be in the form of Dover's powder, or other medicines that determine to the skin, and alternated with castor oil, or other mild laxative. The diseases of children are almost all of an inflammatory chaacter—and the removal of inflammatory action by proper depletion is the best mode of conquering excitement.

II. LOCAL AND GENERAL BLEEDING. Under this head Mr. Marley has made some judicious remarks. In all cases of internal inflammation, of a serious character, he advises general bleeding in children—from the arm in visceral phlogosis—from the external jugular, or from the temporal artery, in cerebral inflammation. Mr. M. has seldom found any difficulty in opening the jugular vein, however young the child; but in infants under 12 or 15 months, it is often difficult to open the veins of the arm. By immersing the member in warm water, the facility of the operation is increased. When these measures are not adopted, or not deemed advisable, he recommends cupping in preference to leeches. His objections to leeches are urged, we think, too strongly; and the great preference of cupping is not very consistent with the following passage.

"I have known considerable nervous excitement produced in children by cupping (particularly on the chest), and occasionally even in adults. I have likewise known extensive local inflammation produced by this operation, but I have never known it end in suppuration."

We have often seen the blaze of the spirit, the pressure of the glass, and the stroke of the scarificator occasion a wince in men of strong minds—and we cannot reconcile to our minds this lavish praise and recommendation of cupping in cases of infantile disease. In children, the feelings are every thing, and the reasoning powers nothing. We have seen the application of cupping-glasses induce instant convulsions, and such a prejudice excited against a practitioner in the minds of the parents, that he was never afterwards employed in the same family. The feelings of the community are not to be outraged or trifled with—and especially when we are urging measures that are really not more effectual, though far more unpalatable than others of a milder character. Nothing is more common than to see practitioners, who are deficient of tact and discretion, ordering a poor person who can scarcely procure bread for his family, to give half a guinea to a cupper, when half a dozen of leeches, costing a couple of shillings, would be equally beneficial, and much less formidable.

III. CROUP. So much does Mr. Marley dread this disease, that whenever he meets with a "child labouring under cold, if it be accompanied by a dry hoarse cough, with pain and difficulty of breathing he very generally has recourse to the measures used for croup"—namely, abstraction of blood from the jugular vein—an emetic—and then a dose of calomel. To this practice in real croup, we do not object; but whether the emetic plan is the most proper for a sharp attack of pulmonic inflammation, we have some doubts.

More than a third of the volume is occupied with the subject of cutaneous diseases, and the execution of the whole is respectable. Mr. Marley's remarks are almost entirely practical, being founded on observations made at the bedside of sickness, rather than drawn from books. This is, perhaps, the best recommendation of the work.

